# Comparing the influence of ‘describing findings to the examiner’ or ‘examining as in usual practice’ on the students’ performance and assessors’ judgements during physical examination skills assessment

**DOI:** 10.15694/mep.2020.000018.1

**Published:** 2020-01-21

**Authors:** Catherine Stephenson, Peter Yeates, Janet Lefroy

**Affiliations:** 1 Keele University School of Medicine

**Keywords:** Objective Structured Skills Examination protocol, Assessment of clinical examination skills, Communication with examiners in OSCEs, Cognitive load in OSCEs

## Abstract

This article was migrated. The article was marked as recommended.

Background:

Within assessment of physical examination skills, two approaches are common: “Describing Findings” (students comment throughout); and examining as “Usual Practice” (students only report findings at the end). Despite numerous potential influences on both students’ performances and assessors’ judgements, no prior studies have investigated the influence of either approach on assessments.

Methods:

Two group, randomised, crossover design. Within a 2-station simulated physical examination OSCE, we manipulated whether students “described findings” or examined as “usual practice”, collecting 1/. performance scores; 2/. Students’/examiners’ cognitive load ratings; ratings of the 3/. fluency and 4/. completeness of students’ presentations and 5/. Students’ task-finishing, comparing all 5 end-points across conditions.

Results:

Neither students’ performance scores nor examiners’ cognitive load were influenced by experimental condition. Students reported higher cognitive load (7/9) when “describing findings” than “usual practice” (6/9, p=0.002), and were less likely to finish (4 vs 12, p=0.007). Presentation completeness was higher for “describing findings” (mean=2.40, (95CIs=2.05-2.74)) than “usual practice” (mean=1.92 (1.65-2.18),p=0.016), whilst fluency ratings showed a similar trend.

Conclusions:

The decision to “Describe Findings” or examine as “Usual Practice” does not appear neutral, potentially influencing students’ efficiency, recall and (by inference) learning. Institutions should explicitly select one option based on assessment goals.

## Introduction

Assessment of clinical skills in medical education relies on direct observation of students and trainees as they perform clinical tasks in either simulated or clinical environments ( Wass *et al.*, 2001). In both Objective Structured Clinical Exams (OSCEs) ( Newble, 2004) and workplace based assessments ( Pelgrim *et al.*, 2011), assessors are instructed to observe trainees closely as they perform tasks in order to form judgements and provide either scores, narrative feedback, or both ( Schuwirth *et al.*, 2011). Implicit to these instructions is the limitation that assessors can only observe that which is observable, or, put differently, performance which can be seen or heard. This may work well for consultation skills where the entirety of a consultation may reasonably be observed, but is more difficult when assessing physical examination or procedural skills. In these latter skills, an assessor may observe that a trainee uses a stethoscope, places their fingers on the patient’s wrist, or gazes towards the patient’s neck, but they cannot observe whether the student hears a wheeze, feels a pulse, or notes the absence of the first jugular venous pulsation. Assessing the recognition of these physical signs (or analogous features of procedural skills assessments) requires either that assessors make inferences from that which is observable or that trainees report their observations as they proceed.

Within assessment of physical examination and procedural skills, two approaches are commonly employed. In the first, students proceed “as in usual clinical practice”, that is, they speak to the patient in order to conduct the task, but they don’t converse with the assessor until the task is complete. This requires that assessors make inferences about the meaning of students’ actions whilst the task proceeds, whilst if students forget to report a finding at the end, the examiner may be unaware of something that the student observed, felt or heard. Assessors making inferences about performance has been implicated as a contributor to unwanted variability in assessors’ judgements ( Kogan *et al.*, 2011), whilst forgetting to report signs may undervalue competent recognition of physical signs ( Williams, Dunnington and Klamen, 2005). Additionally, whilst examining under “usual practice” conditions, students have the option to update their interpretation of equivocal findings in light of the collective whole and thereby achieve the appearance of better physical sign recognition than they actually possess.

In the second approach, students “describe their findings” concurrently whilst they examine, verbalising the things they feel, hear or observe for the examiners benefit. Whilst this prevents the need for examiners to make inferences, and commits students to the interpretation of their findings, it is highly clinically inauthentic, which could reduce the validity of the assessment ( Kane, 2013). Moreover, verbalising may be presumed to impose additional mental workload ( Norman, 2013) on both students as they perform the task, and assessors as they listen and observe. For students this might interfere with task performance ( van Merriënboer and Sweller, 2005) reducing how well they perform, but could also aid encoding of observations into episodic memory ( Tulving, 2002) which might enable them to be reported back more completely or fluently later. For examiners, “describing findings” could contribute to an already high mental workload ( Byrne, Tweed and Halligan, 2014), which is known to influence both examiner variability and attention ( Tavares and Eva, 2014; Tavares, Ginsburg and Eva, 2016), which could increase unwanted examiner variability.

Given the complexity and potential implications of this issue, it is surprising that it is rarely considered. Indeed, whilst some published advice briefly considers this issue ( Cascarini and Irani, 2005), we have not been able to find research which considers the influence of either “describing findings” or examining as in “usual practice” on the process or outcomes of assessment.

Despite this paucity of directly related prior research, some useful parallels may potentially be drawn from research in psychology and related fields on the use of “think-aloud” methods ( Ericsson and Simon, 1980). While usually used in research (rather than assessment) settings, this technique involves participants describing their thoughts either whilst conducting a task (concurrent think aloud) or immediately after completing the task (retrospective think aloud). Prior research suggests that concurrent think aloud imposes additional cognitive load on participants ( Russo, Johnson and Stephens, 1989), and tends to slow task completion ( Ericsson and Simon, 1998). The latter may be particularly relevant to OSCEs, as time for tasks is strictly limited, so an influence which slows performance may reduce task completion. Studies which have examined whether concurrent think aloud interferes with task performance have shown varied findings across contexts ( Fox, Ericsson and Best, 2011) with authors suggesting that this depends on the complexity of the task and how readily thoughts are verbalised ( Russo, Johnson and Stephens, 1989; Ericsson and Fox, 2011). Retrospective think aloud is recognised to provide less complete accounts ( Kuusela and Paul, 2000; Taylor and Dionne, 2000), suggesting that forgetting could potentially limit students’ reporting of findings in the manner we have suggested.

Given the potential importance of this issue to assessment, and limits to the applicability of findings from think aloud research to an assessment context, we aimed to investigate the influence of students “describing” their positive and negative findings versus proceeding as in “usual practice” on assessment processes and outcomes, by addressing four specific hypotheses:

H1: Describing will reduce students’ performance scores compared with usual practice.

H2: Describing will increase examiners’ and students’ cognitive load scores compared with usual practice.

H3: A greater proportion of students will fail to finish physical examinations when describing as compared with usual practice.

H4: Describing will influence ratings of the fluency and completeness of students’ presentations of their findings.

## Methods

### Overview

We used a two-group, randomised, crossover experimental design, collecting data from a simulated two station physical examination skills OSCE. Stations tested respiratory and abdominal examination. Counter-balancing meant that students performed one station under each experimental condition, whilst examiners examined the same station under both conditions for different students.

### Population, sampling and recruitment

The study population was Year 3 undergraduate medical students at Keele University School of Medicine. Sampling was from two successive year groups, with recruitment via email invitation sent to the whole cohort. Data were collected in two separate iterations using the same protocol. The first iteration was in June 2017, when students had evolving clinical experience and had undertaken the Year 3 OSCE which requires they examine as in “usual practice”. The second iteration took place in February 2018, when the following cohort of students had not yet performed their year 3 OSCE, so were more familiar with the “describing findings” format which is required in Years 1 & 2 of the course. As a result, students’ familiarity with the two study conditions was balanced across iterations of data collection. Students in the two cohorts had followed the same course since entry to medical school, with an introduction to clinical examination and limited practical placements in year 2 followed by transition to ward based learning in year 3, with attendant greater exposure to patients and opportunities for “real life” practice.

All participants provided informed written consent. Ethical approval for the study was granted by Keele University Research Ethics Committee (ref ERP1327)

### Procedure

Within iteration 1, participants were randomised into 2 groups. All undertook both OSCE stations in the same order (respiratory examination then abdominal examination), but group 1 performed the respiratory exam whilst “describing findings” and the abdominal exam as in “usual practice”, whereas group 2 performed the respiratory exam as in “usual practice” and the abdominal exam whilst “describing findings”. There were two examiners per station, who remained constant for all students and resultantly examined their station under both study conditions.

**Figure 1.**
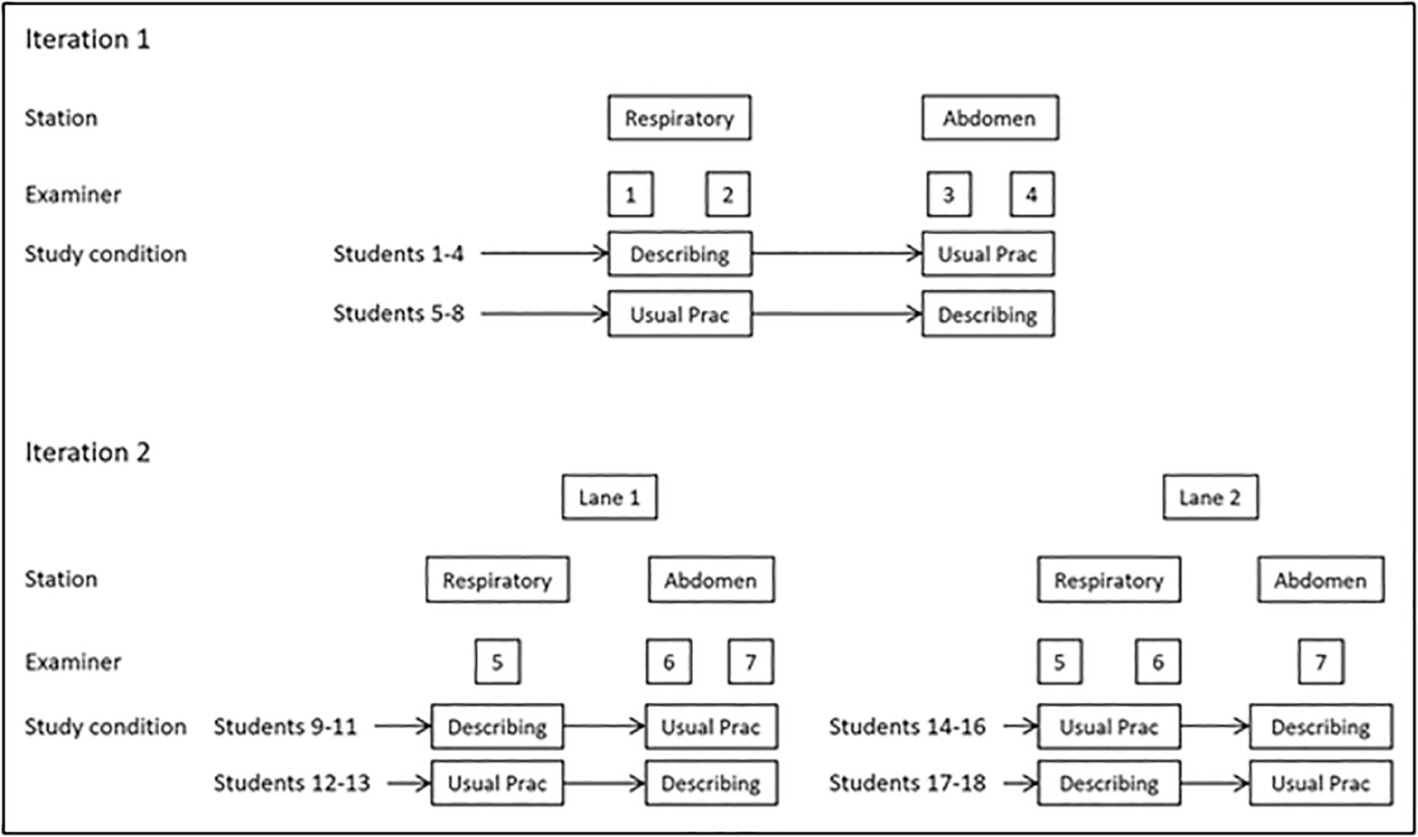
Study design

For iteration 2, students were randomised to 2 parallel circuits of the simulated OSCE before further randomisation to the same two groups as in iteration 1. Participants performed both stations in the same order, and the study condition (describing or usual practice) was manipulated in the same manner as in iteration 1. Owing to examiner availability, in the 1 ^st^ parallel circuit, there were 2 examiners for the respiratory station and 1 for the abdominal station, whilst for the 2 ^nd^ parallel circuit there was 1 examiner for the respiratory station and 2 examiners for the abdominal station. As for iteration 1, examiners were constant for all students in their circuit, and as a result all examiners examined their station under both conditions. Details of the iterations and experimental conditions are illustrated in figure 1.

Resultantly, the main study manipulation (describing findings or usual practice) was counterbalanced across iterations, stations, students and examiners. Station information clearly stated whether students were to describe their findings or perform as in usual practice. Researchers re-iterated these instructions to students immediately before entering stations and alerted examiners when the condition changed.

### Station details

Standard station equipment was used. Simulated patients were recruited from Keele School of Medicine’s “Patients as Educators” scheme and all had demonstrable physical signs. Stations were electronically timed. Students spent 8 minutes 30 seconds in each station with a further 90 seconds to move stations and read instructions. Regardless of study condition, all students were prompted to report their findings to the examiner(s) during the final two minutes of the station. Stations were video recorded. Different patients were used for the first and second iterations.

### Examiner selection and scoring format

All examiners were experienced medically qualified undergraduate OSCE examiners who had previously undergone examiner training and benchmarking. Examiners were provided with station-specific descriptors of good performance, and scored performances using Keele’s Generic Consultation Skills (GeCoS) domain-based assessment instrument ( Lefroy *et al.*, 2011). Student performances were scored for each of 4 domains: communication, examination technique, examination findings and case presentation, on a 4 point scale of: 1 “must improve”, 2 “borderline”, 3 “proficient” and 4 “very good”. Examiners were asked to provide written free-text feedback to each student on their performance. Examiners personally examined the volunteer patients, producing a “gold standard” list of the findings.

### Cognitive Load ratings

Immediately after each student’s performance in each station, both the examiner(s) and student completed a 9 point cognitive load scale ( Paas *et al.*, 2003), to indicate their own perceived task-related cognitive load. In this scale, 1 represents very very low mental effort and 9 very very high mental effort.

### Review of videos to determine fluency and completeness of presentations

In order to determine the fluency and completeness of presentations, videos were segmented by a technician to display (only) students’ presentations of their finding. Two researchers (CS, JL, both highly experienced educators and examiners), blinded to study condition, independently reviewed each presentations, rating them for fluency and completeness. Completeness was rated relative to the “gold standard” findings on a 4 point scale: 1, few key findings, some false findings; 2, some key findings and some false findings; 3, most key findings and no false findings or all key findings and some false findings; 4, all key findings no false findings. Fluency was rated based on CS and JL’s professional judgement on a 4 point scale: 1 being “must improve”, 2 “borderline”, 3 “proficient” and 4 “very good”.

### Judgement of Task completion

As task completion required a less subjective judgement, one researcher (CS) subsequently watched videos unblinded to determine whether students a/. finished the examination during the allotted time, or b/. did not finish the examination in the allotted time.

### Dependent variables and Analysis

Six variables were calculated to address our hypotheses. Firstly, we averaged each student’s scores across the 4 domains in each station to give a single performance score from each examiner. Where two examiners had scored the same performance, we used the mean of their individual scores for that performance. This gave us the first dependent variable: “performance scores”. As prior research has shown that collapsed Likert scales are adequate for parametric analyses ( Carifio and Perla, 2008), we treated this as an interval variable. Next we calculated the average of examiners’ self-rated cognitive load scores (where scores were paired) or used the individual examiner’s rating. This gave us the “examiner cognitive load” variable for each student performance which we treated as ordinal. Similarly we used students’ individual ratings of cognitive load for each performance (“student cognitive load”), which we also treated as ordinal.

Next we averaged across the independent fluency and completeness ratings provided by the two blinded analysts for each student performance to give the interval variables of “fluency” and “completeness” for students’ presentations for each performance.

Finally we recorded the categorical judgement (finished task vs did not finish task) from the assessment of whether students finished the examination task during the allotted time.

To accommodate the study’s crossover design we used Generalized Linear Modelling with Generalized Estimating Equations (GLM GEE) to test hypotheses. We used a subject variable of “students”, with within-subjects variables of “station” (respiratory or abdominal examination) and “describing” (“describing findings” or “usual practice”). We included a between-subjects variable of “iteration” (June 2017 or February 2018 data collection). As examiners were fully nested within iteration and station, and counterbalanced across conditions, we didn’t model examiners as a factor. We examined for main effects of station and iteration during each analysis, and where these were statistically significant we repeated the analysis examining the interaction of “describing” and the significant variable, and any subsequent influence on the comparisons of interest. We sequentially substituted each of the 5 dependent variables into this model, switching between linear and logistic models depending on the variable. GLM GEE analyses produce a “Wald Chi-squared value” (Wald χ ^2^)which is analogous to the *F* statistic in analysis of variance. We have reported this along and the p-value at the relevant points in the text. Where multiple comparisons were performed within the same hypothesis, we adjusted our significance threshold accordingly using the Bonferroni correction for multiple comparisons. The GLM GEE model was not able to accommodate the binary outcome of “Task Finished” vs “Task Not Finished”, so instead we compared the frequency of “finishing” or “not finishing” outcomes for conditions of “describing” or “usual practice” using a Fisher’s exact test. Whilst this ignored the data structure, other factors (iteration and station) were balanced across both conditions. We calculated statistical power of our primary end-point (performance scores) based on a simple 2-mean, 2-sided Student’s t-test, with β=0.80, α=0.05, and a minimally important difference of 0.5 out of 4.0. Main analyses were conducted in IBM SPSS version 21; power was calculated using an on-line power calculator (Brant, 2019).

## Results/Analysis

### Overview

Two hundred and forty-nine students were approached (116 (2017), 133 (2018)). Twenty five expressed an interest of whom 18 participated. All were randomised and all completed the protocol, giving 36 OSCE performance scores for analysis (18 under each study condition). One student withdrew consent for inclusion of their videos so only seventeen students’ presentations of findings (total 34 presentations) were rated for fluency and completeness. No other data were missing.

Table 1 contains a summary of the results. Performances scores were normally distributed, with a mean of 2.77 out of 4.0 and standard deviation of 0.54. Performance scores did not differ by station: respiratory examination mean 2.84 (95% CI 2.58 - 3.10); abdominal examination mean 2.70 (95% CI 2.44 - 2.96), *F*(df)=0.59(1), p=0.45. Performance scores varied by iteration, with students in iteration 1 (mean 2.46 (95% CI 2.22 - 2.70) showing lower scores than students in iteration 2 (mean 3.02 (95% CI 2.81 - 3.23, *F* (df)=12.6(1) p=0.001). Students’ cognitive load scores had a median of 6 (IQR 1) out of 9 whilst examiners’ cognitive load scores had a median of 5.5 (IQR 0.5) out of 9. Collapsed ratings (i.e. the mean of the two independent ratings) of the fluency and completeness of students’ presentations were both normally distributed with means (and standard deviations) of 2.53 (0.83) out of 4.0 for fluency and 2.16 (0.85) out of 4.0 for completeness.

### Hypothesis 1: Describing will influence students’ performance scores

Our first hypothesis posited that the “describing findings” condition would reduce students’ performance scores compared with the “usual practice” condition. Consistent with our descriptive results, the influence of station was not significant (respiratory examination mean = 2.80 (95% C.I.s 2.59 - 3.03), abdominal examination mean = 2.67 (2.48-2.86) Wald χ ^2^=0.70, p=0.40), whereas the influence of iteration of the study was significant (iteration 1 mean score = 2.46 (2.27-2.64), iteration 2 mean score = 3.02 (2.85-3.19) Wald χ ^2^=18.6, p<0.001). There was no significant difference in performance scores between the “describing” and the “usual practice” condition (describing condition mean score = 2.68 (2.47-2.90), usual practice mean score = 2.79 (2.60-2.99), Wald χ ^2^=0.45, p=0.50). Given the SD=0.54, and n=18 we had 79.4% power to detect a difference of 0.5 out of 4.0 in performance scores between conditions. Repeating the model including the interaction of “iteration” x “describing condition” showed that this interaction was non-significant (Wald χ ^2^=0.46, p=0.50) and changed the model parameters very little. As a result there was no evidence to support hypothesis 1. Students’ performance scores were not influenced by whether they described their findings or performed as they would in clinical practice.

### Hypothesis 2: Describing will increase examiners’ and students’ cognitive load scores

Our second hypothesis posited that the “describing” condition would increase examiners’ and students’ cognitive load scores compared to the “usual practice” condition. Examiners’ cognitive load differed by station (respiratory examination median = 5.5 (IQR=0.5), abdominal examination median = 5.0 (0.6) Wald χ ^2^= 8.46, p=0.004), whereas there was no influence of iteration on examiners’ cognitive load (Wald χ ^2^= 1.19, p=0.27). There was a trend towards significance for the “describing findings” condition (describing median examiner cognitive load = 5.25 (IQR=0.6), usual practice median examiner cognitive load = 5.50 (IQR=0.6) Wald χ ^2^= 2.86, p=0.091), although following Bonferroni correction of the significance level to p=0.025 this parameter was non-significant. The interaction of “describing” x station was not significant (Wald χ ^2^=1.06,p=0.30) and its inclusion in the model only minimally altered the resulting parameters.

Students’ cognitive load was not influenced by station (Wald χ ^2^=2.25,p=0.13) or iteration of the study (Wald χ ^2^=0.58,p=0.45), but students’ cognitive load scores were significantly higher for the describing condition (median 7 IQR 2) than for the usual practice condition (median 6, IQR 0, Wald χ ^2^=9.71, p=0.002) which remained strongly significant after within-hypothesis Bonferroni correction of the significance level to 0.025, to account for multiple comparisons. As a result Hypothesis 2 was partly supported in that students’ cognitive load scores were increased by the describing condition, but examiners’ cognitive load scores were not.

### Hypothesis 3: A greater proportion of students will fail to finish physical examinations when describing as compared with usual practice.

Our third hypothesis posited that the describing condition would increase the number of students who did not finish the physical examination task, compared with the usual practice condition. In the describing condition, across both stations, 4 students finished their physical examination, while 13 students did not finish; in the usual practice condition, across both stations, 12 students finished the physical examination task, while 5 did not finish. This difference was statistically significant χ ^2^(df)=7.56 (1),p=0.007.

### Hypothesis 4: Describing will influence ratings of the fluency and completeness of students’ subsequent presentations of their findings.

Our fourth hypothesis posited that blinded ratings of the fluency and completeness of students’ presentations would be influenced by the describing condition compared with the usual practice condition. Neither the station (Wald χ ^2^=0.013,p=0.90) nor the iteration of the study (Wald χ ^2^=3.18,p=0.075) influenced ratings of the fluency of presentations, although as the latter showed a trend towards significance we included its interaction with the “describing” condition in the model which was non-significant (Wald χ ^2^=0.01,p=0.91) and changed the findings only minimally. There was a trend towards significance for the influence of the “describing” condition (mean rating = 2.66 (95% CIs 2.25-3.07) compared to the “usual practice” condition (mean rating = 2.37 (2.08-2.65), Wald χ ^2^=3.81,p=0.051), although this is further from the within-hypothesis Bonferroni corrected significance level of 0.025.

Ratings of the completeness of students’ presentations were influenced by station (respiratory examination mean completeness rating = 1.85 (95% CIs 1.49-2.21), abdominal examination mean completeness rating = 2.44 (2.15-2.73), Wald χ ^2^=7.48, p=0.006) and by iteration of the study (iteration 1 mean completeness rating = 1.88 (1.56-2.19), iteration 2 mean completeness rating = 2.42 (2.02-2.82), Wald χ ^2^=4.38, p=0.036). Consequently the interactions of both terms with the “describe” condition were included in the model, but neither was significant (station x “describe” Wald χ ^2^=3.08,p=0.079; iteration x “describe” Wald χ ^2^=1.87,p=0.17. Blinded ratings of the completeness of students’ presentations were higher when students had described their findings (mean completeness rating = 2.40 (2.05-2.74) compared with when students performed as they would in usual practice (mean completeness rating = 1.92 (1.65-2.18), Wald χ ^2^=5.80,p=0.016). This p value was below the within-hypothesis Bonferroni-corrected significance level of 0.025. As a result there was partial support for hypothesis 3: ratings of the completeness of students’ presentations were higher under the describing condition than under the usual practice condition, whilst there was a trend towards the rating of the fluency of students’ presentations also being higher under the describing condition than under usual practice conditions.

**Table 1.** Summary of hypothecated results

	DescribingFindings	UsualPractice	Wald χ ^2^	p	α level
Performance scores (95% C.I.)	2.68 (2.47-2.90)	2.79 (2.60-2.99)	0.45	0.50	0.05
Examiners’ cognitive load (Inter-quartile range)	5.25 (0.6)	5.50 (0.6)	2.86	0.091	0.025
Students’ cognitive load (Inter-quartile range)	7 (2)	6 (0)	9.71	0.002	0.025
Fluency of presentations (95% C.I.)	2.66 (2.25-3.07)	2.37 (2.08-2.65)	3.81	0.051	0.025
Completeness of presentations (95% C.I.)	2.40 (2.05-2.74)	1.92 (1.65-2.18)	5.80	0.016	0.025
	Describing Findings	Usual Practice	χ ^2^	P (Fishers exact)	α level
Finished Task	4	12	7.56	0.006(0.007)	0.05
Did Not Finish Task	13	5			

## Discussion

### Summary of results

This study examined the influence of asking students to “describe their findings” or examine as in “usual practice” whilst performing physical examination in a simulated OSCE. Contrary to expectations, we found no influence of the instruction to describe findings on either students’ performance scores or on examiners’ cognitive load scores, although students were more likely to fail to finish the physical examination task when describing. Conversely (and as predicted), we found that “describing findings” increased students’ cognitive load scores compared with usual practice, whilst also increasing ratings of the completeness of their presentations, suggesting a possible increase in the fluency of their presentations.

### Theoretical implications

Students’ performance scores were influenced by the iteration of the study, with students in iteration 1 (who were at the end of year 3) scoring lower than students in iteration 2 (who were at the beginning of year 3 in the next cohort). Whilst counter intuitive this may be explained by the timing of data collection: iteration 1 was just before resit OSCEs, and may therefore have recruited less able students who were resitting whereas iteration 2, at the start of a new semester, may have recruited highly motivated students.

Several other theoretical considerations emanate from these findings. Most importantly there was no influence of study condition (“describing findings” or “usual practice”) on students’ performance scores. At a theoretical level this may be surprising. Consistent with our findings, verbalising thoughts is expected to both increase mental workload and slow task performance ( Ericsson and Simon, 1980). The observation that describing findings increased mental workload and reduced task completion, without affecting performances scores warrants some consideration. This suggests that examiners’ domain-based judgements were predicated on observations of what students did, and how they did it, during the observed performance more than whether they finished the task.

It is generally accepted that cognitive resources are fixed ( Baddeley, 1992), and when they are exceeded, performance is impaired ( Mousavi, Low and Sweller, 1995). Our observation of unimpaired performance under conditions of increased cognitive load suggests that the task did not exceed students’ maximal mental workload under either condition, or put differently that they had enough spare resources to manage the extra cognitive load of verbalising on top of performing physical examination tasks. As a result, these findings don’t exclude the possibility that students’ performance might be impaired by “describing findings” in a more cognitively demanding station.

The increased cognitive effort which students exerted is particularly interesting if considered through the lens of “Cognitive Load Theory” ( Sweller, 1988). This theory posits that learning tasks involves “intrinsic load” (effort exerted on completing the task itself), “extrinsic load” (effort imposed by unnecessary elements of the task - for example collating information from different sources), and “germane load” (effort expended on making links and developing schema). Intrinsic load is usually inherent to the task and can’t be altered, whilst extrinsic load is usually imposed by features of the way the task is designed. Increasing extrinsic load generally reduces task performance ( Paas *et al.*, 2003), whilst conversely increasing germane load increases overall cognitive load but despite this results in better task performance and knowledge retention ( Stark *et al.*, 2002). “Describing findings” increased cognitive load, and, whilst it had no influence on overall performance, it increased the completeness of presentations of findings. As such it may have functioned as a form of germane load; it made the task harder work but benefitted students’ ability to report findings, probably through a form of rehearsal, which made them subsequently easier to retrieve when presenting ( Roediger, 2008). As a result “describing findings” may have the potential to benefit learning, through cognitive elaboration of the task ( Rikers, Schmidt and Boshuizen, 2002). For the purposes of assessment, however, this improvement in students’ ability to report findings when they have already described the findings during their examination risks over-representing of students’ ability to present a case in clinical practice. In clinical practice doctors are expected to retain and process the information to present.

### Considerations for practice

Further work is needed to replicate and extend these findings before any firm suggestions can be made for practice. Nonetheless a few potential practical implications can be considered. Pertinently, within performance assessments in medical education, instructions to either “describe findings” or examine as in “usual practice” are not neutral and as a result it seems inadvisable to simply leave this instruction to examiners’ whim ( Cascarini and Irani, 2005). Consequently, we recommend that assessment designers clearly stipulate one instruction or the other in their guidance and assessor training. Extra time might also need to be factored into OSCE stations if students are expected to describe findings.

If these findings can be confirmed, we suggest that “describing findings” and performing as in “usual practice” may have different applications at different stages in learning. The added germane load of “describing findings” may produce a useful benefit for students’ learning during initial learning or observed practice of procedures or physical examination skills, and as a result it may be more useful in a coaching scenario ( Watling and Ginsburg, 2019). Conversely, the clinical inauthenticity of “describing findings”, combined with the potential for it to enhance presentation of findings may mean that performing as in “usual practice” produces inferences with greater validity for summative purposes ( Downing, 2003) than “describing findings”. As a result consideration may be given to shifting approach depending on the purpose of the assessment or learning situation.

### Strengths and Limitations

There are several strengths to this study. To enable unbiased measurement of the effect of “describing findings” on students and examiners we used a randomised, controlled, experimental design, addressing carefully theorised pre-specified hypotheses. Despite these strengths it has some weaknesses. The generalisability of the findings are limited by the study’s sampling, from students at a particular stage in the course at one medical school; students who are familiar with different assessment systems could have produced different results. This is partly mitigated by the fact that we included groups of students who were both 1/. accustomed and 2/. relatively naive to the “describing findings” condition. Our study was powered to find a difference in performance scores of 0.5 out 4.0 between conditions; we can’t exclude the possibility that smaller differences could have occurred. We only sampled students at a single level of training; other effects may exist for more sophisticated clinical learners. Whilst we sampled across two very common physical examination tasks, we can’t exclude that other effects might have emerged for more complex scenarios or for procedural skills. Lastly we used a self-reported rather than an objective measure of participants’ cognitive load. Whilst objective measurement may have benefitted the internal validity of the study, procedures required for objective cognitive load measurement ( Klingner, Kumar and Hanrahan, 2008) would have reduced the ecological validity of our findings.

### Recommendations for future research

Future research should seek to replicate these findings across contexts and for other scenarios and groups of learners, in order to determine their universality within assessment. Further work might compare effects of “describing findings” and “usual practice” on the accuracy of examiners’ observations as compared to an objective measure (i.e. video-based checking of performance or via simulation manikins where findings can be controlled), in order to determine whether “describing findings” does or does not lead to better observation of physical sign recognition by assessors.

## Conclusion

Although the performance scores did not show a difference between “describing” and “usual practice” the assessment decision to ask students to either “describe their findings” or examine as in “usual practice” is not neutral, influencing both cognitive intermediary and some outcome level processes. Institutions should reflect on this choice, making it explicit through guidance and assessor training, and allowing more time for task completion if “describing” is the preferred format. Whilst further work is required, we can recommend “describing findings” if the aim is learning but asking students to examine as in “usual practice” in summative assessments.

## Take Home Messages

• Describing findings to the assessor while examining the patient in an OSCE station did not significantly affect the students’ scores.
• Student cognitive load was increased and task completion reduced by describing, but subsequent presentation of findings was more complete when students had described while examining.
• When designing assessments, institutions should be explicit as to whether candidates are to “describe findings” or examine “as in usual practice” dependent upon the purpose of the assessment.

## Notes On Contributors

Catherine Stephenson is a teaching fellow, lead for systems examination teaching at Keele University School of Medicine and a GP.

Peter Yeates is a lecturer in medical education research and a consultant in acute and respiratory medicine. His interests include assessor cognition research, psychometrics and innovative ways to increase OSCE standardisation.

Janet Lefroy is a senior lecturer in medical education, lead for consultation skills teaching at Keele University School of Medicine and a GP. Her interests include assessment of consultation skills, feedback to clinical trainees on their skills, and the transition between medical school and the clinical workplace.
