# Supplementary material for: Comparing the influence of ‘describing findings to the examiner’ or ‘examining as in usual practice’ on the students’ performance and assessors’ judgements during physical examination skills assessment
Source: MedEdPublish (2016). 2020 Jan 21;9:18. [Version 1] doi: 10.15694/mep.2020.000018.1 (PMC10702660; doi:10.15694/mep.2020.000018.1)
Supplement: Peer review history

# Reviewer report: Reviewer response for version 1

**DOI:** 10.21956/mep.19964.r30340

**Published:** 2020-01-26

**Authors:** Ian Wilson

**Affiliations:** 1 International Medical University

This review has been migrated. The reviewer awarded 5 stars out of 5 This is a well researched paper of practical significance to educators and assessors. In line with the authors' recommendations this study needs to be replicated in other settings. Personally I would like to see the effect of years of training on the outcome.Welldone

Reviewer Expertise:

NA

No decision status is available

# Reviewer report: Reviewer response for version 1

**DOI:** 10.21956/mep.19964.r30337

**Published:** 2020-01-22

**Authors:** Sarah Williams

**Affiliations:** 1 Vanderbilt University Medical Center

This review has been migrated. The reviewer awarded 4 stars out of 5 Great study. Well written and clear. Great guide for future replication.

Reviewer Expertise:

NA

No decision status is available

# Reviewer report: Reviewer response for version 1

**DOI:** 10.21956/mep.19964.r30339

**Published:** 2020-01-22

**Authors:** Mini Singh

**Affiliations:** 1 University of Manchester

This review has been migrated. The reviewer awarded 4 stars out of 5 Thank you for an article that speaks to an important part of the curriculum.i like the straight forward replicable methods

Reviewer Expertise:

NA

No decision status is available

# Reviewer report: Reviewer response for version 1

**DOI:** 10.21956/mep.19964.r30338

**Published:** 2020-01-21

**Authors:** Felix Silwimba

**Affiliations:** 1 University of Lusaka

This review has been migrated. The reviewer awarded 5 stars out of 5 this is a very good study. it has provided some evidence on OSCE process. it should be replicated in other medical schools.

Reviewer Expertise:

NA

No decision status is available

# Reviewer report: Reviewer response for version 1

**DOI:** 10.21956/mep.19964.r30341

**Published:** 2020-01-21

**Authors:** John Cookson

**Affiliations:** 1 University of Worcester

This review has been migrated. The reviewer awarded 4 stars out of 5 Thank you, good to have some evidence in a disputed area. My own policy in summative assessements is to instruct examiners to use 'usual practice' and the students know and expect that.They have enough to do without talking about it and they will not generally have been trained and practiced to do both

Reviewer Expertise:

NA

No decision status is available
